# The Physiological Anatomy and Physiology of Man

**Published:** 1845-07

**Authors:** 


Art. I.—*The Physiological Anatomy and Physiology of Man.* By Robert Bentley Todd, m.d. f.r.s., Professor of Physiology in King's College, London; and William Bowman, f.r.s. Demonstrator of Anatomy in King's College, London. Part II.—*London,* 1845. 8vo, pp. 248. With 50 Wood Engravings.

When we noticed the appearance of the First Part of this work, two years ago, it was with the full expectation that the authors would redeem the pledge which they voluntarily gave to the public, by the speedy completion of the treatise. They then announced that it would be comprised in *two* more parts; and that these were in such a state of preparation, that their publication might be anticipated in the course of the ensuing year. Two years have elapsed, and now the Second Part appears, with the announcement that *two more* parts are yet to come; and warned, it may be presumed, by past experience, they do not promise anything more respecting their time of publication, than that they will follow "at an early period." Now, whatever may be the necessity for adopting this system in the production of an extensive encyclopædiacal work which no publisher would venture to bring out in a complete form, even if an editor could prepare it, the same necessity does not hold in the case of a systematic treatise upon a single subject, within moderate compass, the whole of which, on every account, ought to be in the hands of the reader at once. We think that the public have a claim to be considered, as well as the author; and that it is the duty of the latter, either to keep back his work until the whole is so nearly prepared that the immediate completion of it is almost a matter of certainty, or, if his preparations be less advanced, to sacrifice every other object, if necessary, to the redemption of his pledge.

We can scarcely recall a single instance in which a systematic medical treatise published in parts, has been completed within the time first specified, whilst, on the other hand, the list of unfulfilled pledges of this sort is a pretty long one. For these reasons, we have invariably set our faces against the system; and have determined to give no more than a passing notice to any work, however valuable it might appear, until it should be completed,—except in very particular cases. The importance of the work before us may, possibly, make us break through our rule, in its favour. For the present, however, we shall content ourselves with stating that the Part before us is worthy of that which preceded it, and, consequently, most creditable to its authors; that it is devoted to the nervous system and its dependencies; that it contains the results of a large amount of original research, together with a comprehensive summary of what has been done by others;—and that the illustrations are surpassingly excellent.

